# *IGF2BP2* and obesity interaction analysis for type 2 diabetes mellitus in Chinese Han population

**DOI:** 10.1186/2047-783X-19-40

**Published:** 2014-07-25

**Authors:** Hui-Hui Wu, Nai-Jia Liu, Zhen Yang, Xiao-Ming Tao, Yan-Ping Du, Xuan-Chun Wang, Bin Lu, Zhao-Yun Zhang, Ren-Ming Hu, Jie Wen

**Affiliations:** 1 Department of Endocrinology and Metabolism, Huashan Hospital, Fudan University, No. 12 Wulumuqi Mid Road, Building 0#, Jing'an District, Shanghai 200040, China; 2 Department of Endocrinology and Metabolism, Jing'an District Center Hospital of Shanghai, Shanghai 200040, China; 3 Department of Endocrinology and Metabolism, Xin Hua Hospital, Shanghai Jiao Tong University, Shanghai 200020, China; 4 Department of Endocrinology and Metabolism, Hua Dong Hospital, Fudan University, Shanghai 200040, China

**Keywords:** *IGF2BP2*, Obesity, Type 2 diabetes mellitus, Chinese Han population

## Abstract

**Background:**

The objective of this study was to systematically evaluate the contribution of the insulin-like growth factor 2 mRNA-binding protein 2 (*IGF2BP2*) to type 2 diabetes mellitus (T2DM) and its interaction with obesity to T2DM susceptibility.

**Methods:**

To clarify whether *IGF2BP2* is an independent risk factor for T2DM in Chinese population, we conducted a study with a total of 2,301 Chinese Han subjects, including 1,166 T2DM patients and 1,135 controls, for the genotype of a most common and widely studied polymorphism—rs4402960 of *IGF2BP2*. Genotyping was performed by iPLEX technology. Gene and environment interaction analysis was performed by using multiple logistic regression models.

**Results:**

The repeatedly confirmed association between *IGF2BP2* (rs4402960) and T2DM had not been replicated in this cohort (*P* = 0.182). Interestingly, we found that obese subjects (body mass index (BMI) ≥ 28.0 kg/m^2^) bearing the minor A allele had an increased risk to develop T2DM (*P* = 0.008 for allele analysis and *P* < 0.001 for genotype analysis).

**Conclusions:**

The present study provided data suggesting that the wild C allele of *IGF2BP2* (rs4402960) had a protective effect against T2DM in obese subjects of Chinese Han population.

## Background

Type 2 diabetes mellitus (T2DM) is a complex metabolic disorder characterized by hyperglycemia as a result of pancreatic beta cell dysfunction and insulin resistance. Multiple environmental factors and genetic determinants are considered to be involved in the pathogenesis of the disease. Genome-wide association studies (GWAS) have emerged as a powerful approach for identifying novel genetic variants contributing to the risk of T2DM and further facilitate the elucidation of the genetic etiology of diabetes. Among the variants relating to glucose metabolism revealed by GWAS, insulin-like growth factor 2 mRNA-binding protein 2 (*IGF2BP2*) was the most extensively studied and supposed to be a T2DM susceptibility gene [1-3].

*IGF2BP2* is encoded by the *IGF2BP2* gene which is located on chromosome 3q27. Its strong association with β cell function is established by regulating *IGF2* post-translation [4]. Several variants of the *IGF2BP2* gene were investigated for relationship with T2DM, of which rs4402960 was the most extensively studied. However, due to the ethnic difference in risk allele frequency, the contribution of this common variant to T2DM appears to be race dependent which makes it a highly controversial candidate for T2DM [1-3,5]. To get a thorough understanding of the effect of *IGF2BP2* on the susceptibility of T2DM, we therefore genotyped for *IGF2BP2* rs4402960 in a total of 2,301 Chinese Han individuals in our present study.

Given that environment/lifestyle changes can modify the risk of T2DM, likely contributing factors such as gender, body mass index (BMI), and smoking behavior of subjects to T2DM would also be interesting to investigate [6]. Obesity (BMI ≥ 28.0 kg/m^2^) is especially widely recognized as an independent risk factor for T2DM in Chinese population [7]. Therefore, it would be valuable to conduct a particular analysis for obesity in this cohort. Previous studies provided evidence for gene-obesity interaction in human complex disease [8,9]. Studies also supported the assumption that *IGF2* is strongly associated with obesity [10]. It is evident that obesity is associated with T2DM, and *IGF2* levels in T2DM patients are associated with T2DM. We hypothesize that obesity may modify the association between *IGF2BP2* and T2DM—also called the interaction of *IGF2BP2* and obesity with T2DM. Since some variants are known to affect the risk of T2DM through obesity [11,12], this work aimed to evaluate the interaction effect of *IGF2BP2* and obesity on T2DM susceptibility.

## Methods

### Study population

Individuals enrolled in the cohort, including 1,166 T2DM patients and 1,135 controls, were of Southern Han Chinese ancestry residing in the Shanghai metropolitan area. T2DM patients registered in the analysis were recruited from the Endocrinology and Metabolism outpatient clinics at Fudan University Huashan Hospital in Shanghai. All subjects in the cohort were informed and consented to take part in the study. The protocol was approved by the Ethics Committee of Huashan Hospital affiliated to Fudan University.

### Measurements

The subjects were interviewed for the documentation of medical histories, medications, regular physical examinations, and laboratory assessment of T2DM risk factors. BMI was calculated as the weight in kilograms divided by the square of height in meters. Systolic and diastolic blood pressure (BP) values were the means of two physician-obtained measurements on the left arm of a seated participant.

Peripheral venous blood samples were collected in tubes in the fasting state and 2 h after diet in all subjects. The blood was centrifuged at 3,000 rpm for 10 min for plasma separation and immediately used to measure biomarkers. Fasting plasma glucose (FPG) and postprandial plasma glucose (PPG) were quantified by the glucose oxidase-peroxidase procedure. Serum total cholesterol (TC), triglyceride (TG), high-density lipoprotein (HDL) cholesterol, urea nitrogen (UN), uric acid (UA), and alanine transaminase (ALT) levels were measured by an enzymatic method with a chemical analyzer (Hitachi 7600-020, Tokyo, Japan). Low-density lipoprotein (LDL) cholesterol levels were calculated using the Friedewald formula. The day-to-day and inter-assay coefficients of variation at the central laboratory in our hospital for all analyses were between 1% and 3%.

### Definition

Hypertension (HT) was defined as blood pressure ≥140/90 mmHg or a history of hypertension medication. BMI was classified based on the Chinese criteria: obese [13] (BMI ≥ 28.0 kg/m^2^). The definition of high serum TG was TG ≥ 150 mg/dl, and that of high serum TC was TC ≥ 200 ml/dl. Diabetes was defined according to the 1999 WHO criteria [14]: fasting plasma glucose ≥7.0 mmol/l and/or 2-h plasma glucose ≥11.1 mmol/l in the oral glucose tolerance test (OGTT). All diabetic patients were unrelated and diagnosed after the age of 27 years. Known subtypes of diabetes, such as type 1 diabetes, gestational diabetes, rare forms of T2DM, and secondary diabetes (pancreatitis, hemochromatosis), were excluded based on antibody measurements and inheritance. The nondiabetic unrelated individuals were identified as the control population based on the following criteria: (1) no family history of diabetes, (2) ≥45 years of age, and (3) normal glucose tolerance verified by OGTT. The clinical characteristics of the participants are summarized in Table 1.

**Table 1:** The clinical characteristics of the subjects

**Variables**	**Diabetics**	**Controls**
*N*	1,166	1,135
Age (years)	65.46 ± 10.56	59.09 ± 7.85
Sex (male/female)	456/710	352/783
Height (cm)	160.20 ± 8.64	161.09 ± 7.65
Weight (kg)	64.85 ± 10.71	62.78 ± 10.15
SBP (mmHg)	139.52 ± 19.92	126.44 ± 16.92
DBP (mmHg)	82.88 ± 10.98	80.52 ± 10.13
BUN (mg/dl)	6.08 ± 1.63	5.62 ± 1.37
UA (mg/dl)	0.30 ± 0.08	0.31 ± 0.08
FPG (mmol/l)	8.39 ± 3.03	5.22 ± 0.38
PPG (mmol/l)	15.05 ± 5.34	6.03 ± 1.04
TC (ml/dl)	5.43 ± 1.11	5.35 ± 1.00
TG (ml/dl)	2.00 ± 1.46	1.47 ± 1.06
HDL (mmol/l)	1.29 ± 0.34	1.43 ± 0.36
LDL (mmol/l)	3.10 ± 0.86	3.10 ± 0.79
ALT (IU/l)	28.44 ± 16.08	24.04 ± 13.69
rs4402960 (A/C)	27.03%/72.97%	25.27%/74.73%

*SBP* systolic blood pressure, *DBP* diastolic blood pressure, *BUN* blood urea nitrogen, *UA* uric acid, *FPG* fasting plasma glucose, *PPG* postprandial plasma glucose, *TC* total cholesterol, *TG* triglyceride, *HDL* high-density lipoprotein cholesterol, *LDL* low-density lipoprotein cholesterol, *ALT* alanine transaminase.

### SNP genotyping

Peripheral venous blood samples were collected from all study subjects, and the genomic DNA was extracted from peripheral blood leukocytes by the conventional proteinase K-phenol-chloroform extraction method. A total of 2,301 Chinese Han individuals were genotyped for *IGF2BP2* rs4402960 by using iPLEX (Sequenom, San Diego, CA, USA); the single nucleotide polymorphism (SNP) involved was detected by matrix-assisted laser desorption/ionization time-of-flight mass spectrometry. The genotype distribution was in Hardy-Weinberg equilibrium (*P* > 0.05), and there was a 99.9% genotype concordance rate when duplicated samples were compared across plates.

### Statistical analysis

Continuous variables were detected when the variables followed normal distribution using the Kolmogorov-Smirnov test. Variables that were not normally distributed were log-transformed to approximate normal distribution for analysis. Results are described as mean ± SD or median unless stated otherwise. Differences in variables between T2DM and control were determined by unpaired *t* test. Between-group differences in properties were accessed by *χ*^2^ analysis. Univariate logistic regression was performed to determine variables associated with T2DM and to estimate confounding factors possibly disturbing the relation of BMI and/or *IGF2BP2* to T2DM. Multivariable logistic regression (MLR) was carried out to control potential confounders for determining the independent contribution of variables to T2DM. For interaction analysis, MLR was conducted to include two main variables and their interaction item to determine the interaction effect. In order to better investigate the interaction between BMI and *IGF2BP2* on T2DM, we performed two analyses according to the following variables: the allele and genotype of *IGF2BP2*. Odds ratios (OR) with 95% confidence intervals (CI) were calculated for the relative risk of BMI and/or *IGF2BP2* with T2DM. Results were analyzed using the Statistical Package for the Social Sciences for Windows version 16.0 (SPSS, Chicago, IL, USA). Tests were two-sided, and a *P* value of <0.05 was considered significant.

## Results

### Clinical characteristics of the subjects

The baseline clinical characteristics of the 2,301 subjects are listed in Table 1. There are 456 males and 710 females (mean age, 65.46 ± 10.56 years) in the cases and 352 males and 783 females (mean age, 59.09 ± 7.85 years) in the controls. Diabetic patients had more weight than controls. Systolic blood pressure (SBP), diastolic blood pressure (DBP), FPG, PPG, TC, and TG levels were higher in the cases than in the controls, while the HDL level was lower in the cases. Serum UA, UN, ALT, and LDL levels were similar between the two groups. The minor allele (A) frequency of rs4402960 was 25.27% and 27.03% in the cases and controls, respectively.

### Univariate and multiple logistic regression analyses for diabetes

To estimate the association of various clinical factors and T2DM, univariate logistic regression models were developed to include age, sex, BMI, hypertension, lipid profiles, UA, and SNP (rs4402960) (Table 2). The univariate logistic analyses indicated that age, sex, BMI, hypertension, and TC were significantly associated with T2DM (*P* < 0.05 for all), except for UA and SNP (rs4402960) (*P* = 0.744 and 0.182, respectively). The proportion of T2DM was 48.17% and 62.75% in the low-BMI group and high-BMI group, respectively. In subjects with high BMI (more than 28.0 kg/m^2^), the OR for T2DM was 1.813 (95% CI 1.538–2.136, *P* < 0.001). MLR demonstrated that BMI remained significantly different between the cases and controls after adjustment for potential confounders (*P* < 0.05, data not shown).

**Table 2:** Univariate logistic regression analysis for diabetes

**Variables**	***β***	**S.E.**	***P *** **value**	**OR**	**95% CI**
Age	0.07	0.003	<0.001	1.07	1.07–1.08
Sex	−0.35	0.06	<0.001	0.70	0.62–0.80
BMI	0.60	0.08	<0.001	1.81	1.54–2.14
HT	1.21	0.09	<0.001	3.36	2.83–3.99
TC	0.07	0.03	0.01	1.07	1.02–1.14
TG	0.46	0.03	<0.001	1.59	1.48–1.70
UA	0.03	0.09	0.74	1.03	0.87–1.22
*IGF2BP2*	0.09	0.07	0.18	1.09	0.96–1.25

*IGF2BP2 with SNP* rs4402960, *HT* hypertension, *S.E.* standard error.

### BMI by *IGF2BP2* interaction analysis for diabetes

**Figure 1:**
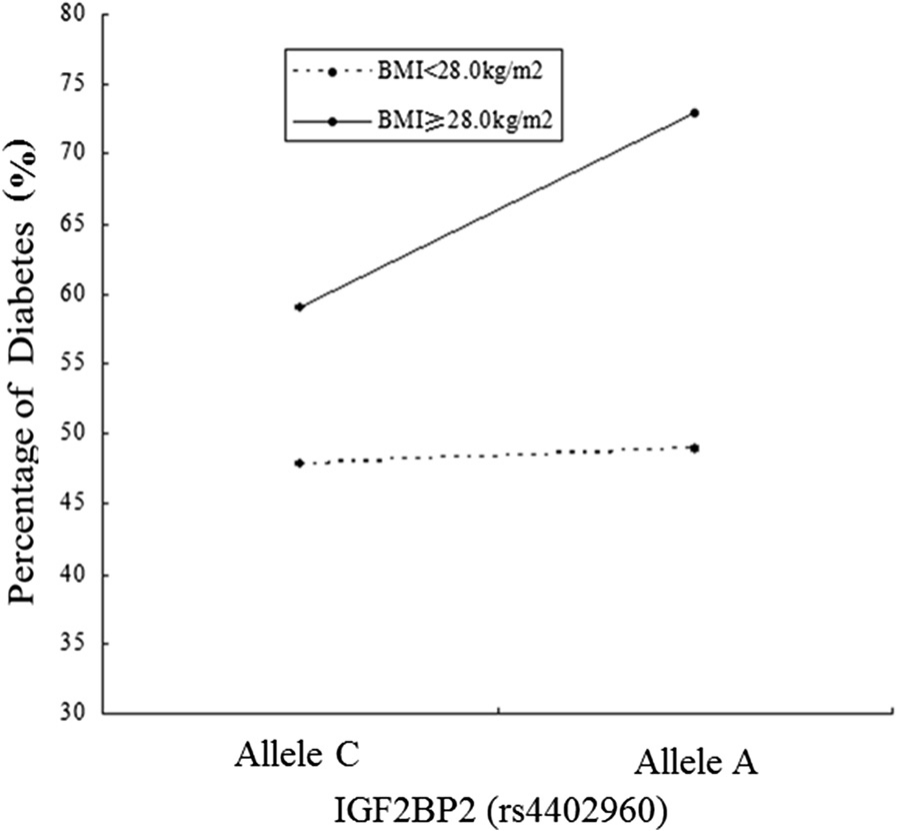
The interaction analysis of BMI and SNP (rs4402960 with the C/A allele) for diabetes.

**Figure 2:**
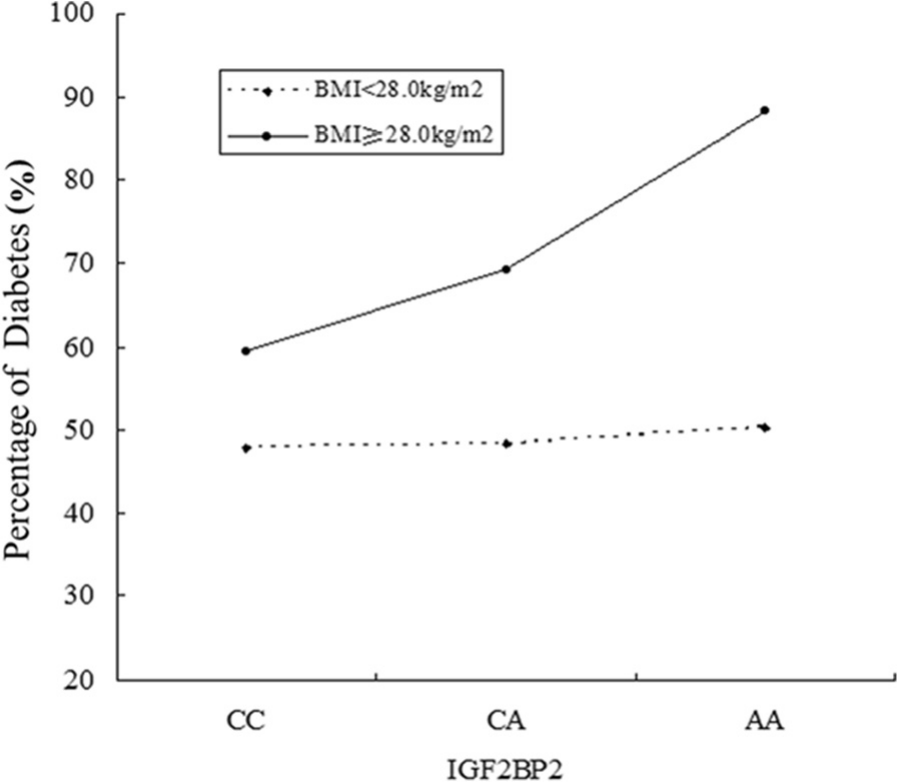
The interaction analysis of BMI and SNP (rs4402960 with the CC/CA/AA genotype) for diabetes.

In the model of rs440296 with the allele variable, MLR models were developed to include two main effect variables: BMI and rs4402960. The interaction item among them was detected in the MLR model after adjustment for relevant potential confounders (*P* = 0.023, Table 3 and Figure 1), and the interaction effect was also estimated (OR_Int_ = 1.603, 95% CI 1.068–2.405). In another model of rs440296 with the genotype variable, the interaction term of BMI and rs4402960 was detected by the same method (*P* = 0.001, Table 4 and Figure 2), as well as the interaction effect (OR_Int_ = 1.661, 95% CI 1.235–2.233). In these two models, MLR models signified that BMI was a significant and independent risk factor for T2DM, while rs4402960 alone was not significantly associated with T2DM. The interaction terms of BMI and SNP rs4402960 which were detected in the two models only confirmed a significant association between this SNP and T2DM in the high-BMI group (*P* = 0.008 for allele analysis and *P* < 0.001 for genotype analysis, data not shown).

**Table 3:** **The interaction effect analysis of BMI and allele of *IGF2BP2 *** **(rs4402690) for diabetes**

**Variables**	***β***	**S.E.**	***P *** **value**	**OR**	**95.0% CI**
BMI	0.53	0.10	<0.0001	1.70	1.40–2.05
rs4402690 (A/C)	0.04	0.07	0.59	1.04	0.90–1.20
BMI by rs4402690	0.47	0.21	0.02	1.60	1.07–2.41

Model adjusted for age, sex, HT, TC, and TG. *S.E.* standard error.

**Table 4:** **The interaction effect analysis of BMI and genotype of *IGF2BP2 *** **(rs4402690) for diabetes**

**Variables**	***β***	**S.E.**	***P *** **value**	**OR**	**95.0% CI**
BMI	0.41	0.11	<0.001	1.50	1.21–1.86
rs4402960 (AA/CA/CC)	0.04	0.05	0.46	1.04	0.94–1.15
BMI by rs4402960	0.51	0.15	0.001	1.66	1.24–2.23

Model adjusted for age, sex, HT, TC, and TG. *S.E.* standard error.

## Discussion

We conducted a study to evaluate the interaction effect of *IGF2BP2* and obesity on T2DM in a large case-control sample of Chinese population. To our knowledge, this is the first investigation of the evaluation of interaction effect on T2DM based on variables of obesity and *IGF2BP2*.

Genotyping was performed for *IGF2BP2* (rs4402960) in a T2DM case-control cohort comprising of 2,301 Chinese Han individuals. However, the documented association of *IGF2BP2* (rs4402960) with T2DM in various ethnic populations [15-18] had not been replicated. In line with our finding, subsequent large-scale population studies also reported a lack of association in Caucasians [19-21]. Such conflicting results of various association studies may be attributed to the diverse ethnic/regional backgrounds, as well as the limited number of participants which had insufficient statistical power to detect a slight effect of the common polymorphism *IGF2BP2* (rs4402960) on T2DM susceptibility. Therefore, a larger sample size is necessary to detect the association between this *IGF2BP2* genetic variant and T2DM.

*IGF2BP2* in pancreatic and adipose tissues can downregulate the expression of IGF2, a growth factor that plays a pivotal role in controlling adipogenesis [22] and pancreatic development [23]. Therefore, *IGF2BP2* may contribute to T2DM through impaired β cell function or alterations in adipose tissue as well. Consistent with the hypothesis, a study observed a more than twofold increase in *IGF2BP2* expression level in the adipose tissue of diabetic patients compared to controls [20]. Similarly, an altered expression of *IGF2BP2* in adipocytes of T2DM subjects compared with healthy people was also detected [24]. Unfortunately, the current study did not find a significant association between *IGF2PB2* (rs4402960) and T2DM, as a consequence of the insufficient statistical power in the present sample size or the different impacts of various *IGF2BP2* genetic variants*.* Further studies are required to elucidate the association of other *IGF2BP2* variants with the risk of T2DM.

Considering that lifestyle changes can modify the risk of T2DM, the likely effect of metabolic quantitative traits on T2DM susceptibility needs to be addressed, as well as their interaction with various gene variants. Here, MLR models were developed to demonstrate that obesity (BMI ≥28.0 kg/m^2^) remained an independently risk factor for T2DM after potential confounder adjustment (*P* < 0.05, data not shown). Although it had been known for decades that both T2DM and obesity have a genetic basis [25], the mechanism by which obesity relates to T2DM in humans is still unclear. Since some genetic variants are known to affect the risk of T2DM through obesity, a hypothesis comes up that *IGF2BP2* may have a relationship with obesity. However, no significant difference in *IGF2BP2* expression in obese patients carrying different rs4402960 genotypes has been found [20].

Our study is the first analysis of the interaction of obesity and *IGF2BP2* variant on T2DM susceptibility. It is noteworthy that the risk imparted by the minor A allele was higher than that by the C allele in obese subjects (*P* = 0.008 for allele analysis and *P* < 0.001 for genotype analysis), thus prompting the speculation of a possible interaction between *IGF2BP2* (rs4402960) and obesity in determining overall T2DM risk. However, the underlying mechanism has been still unknown. The associations between variants of *IGF2BP2* and abdominal/visceral total fat were evidenced in Canadian Caucasians [26] and Mexican Americans [12], suggesting a possible role of *IGF2BP2* in insulin resistance. This finding implies that *IGF2BP2* (rs4402960) may disturb T2DM susceptibility through its contribution to insulin resistance, which is experienced mainly by obese individuals.

Several limitations of the study deserve comment. Firstly, the subjects who participated in the study were recruited from Shanghai, so they may not have been representative of China as a whole. Secondly, it is important to mention that our study was performed on Chinese individuals, and our findings may not be relevant to people of other ethnicities.

## Conclusions

The present study did not replicate the association of *IGF2BP2* (rs4402960) and T2DM. Interestingly, it provided data suggesting that the wild C allele of *IGF2BP2* (rs4402960) had a protective effect against T2DM in obese subjects of Chinese Han population. Further studies and a larger sample size are required to elucidate the relevant mechanisms, as well as the likely contribution of other *IGF2BP2* variants to the risk of T2DM.

## Competing interests

The authors declare that they have no competing interests.

## Authors' contributions

JW and R-MH conceived of the study, participated in its design and coordination, and helped to draft the manuscript. H-HW participated in the design of the study, performed the statistical analysis, and drafted the manuscript. N-JL, ZY, X-MT, Y-PD, X-CW, BL, and Z-YZ contributed the samples, reagents, and analysis tools. All authors read and approved the final manuscript.
